# Calculations of Absolute Solvation Free Energies with Transformato—Application to the FreeSolv Database Using the CGenFF Force Field

**DOI:** 10.1021/acs.jctc.3c00691

**Published:** 2023-08-24

**Authors:** Johannes Karwounopoulos, Åsmund Kaupang, Marcus Wieder, Stefan Boresch

**Affiliations:** † Faculty of Chemistry, Institute of Computational Biological Chemistry, University of Vienna, Währingerstr. 17, 1090 Vienna, Austria; ‡ Vienna Doctoral School of Chemistry (DoSChem), University of Vienna, Währingerstr. 42, 1090 Vienna, Austria; § Department of Pharmacy, Section for Pharmaceutical Chemistry, University of Oslo, 0316 Oslo, Norway; ∥ Department of Pharmaceutical Sciences, Pharmaceutical Chemistry Division, University of Vienna, Althanstrasse 14, 1090 Vienna, Austria

## Abstract

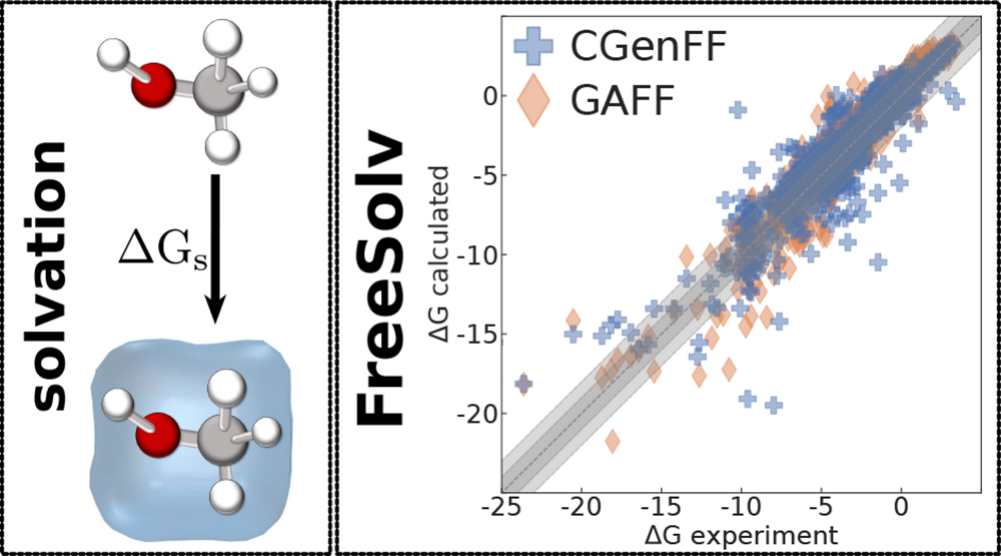

We recently introduced transformato, an open-source Python package for the automated setup of large-scale calculations of relative solvation and binding free energy differences. Here, we extend the capabilities of transformato to the calculation of absolute solvation free energy differences. After careful validation against the literature results and reference calculations with the PERT module of CHARMM, we used transformato to compute absolute solvation free energies for most molecules in the FreeSolv database (621 out of 642). The force field parameters were obtained with the program cgenff (v2.5.1), which derives missing parameters from the CHARMM general force field (CGenFF v4.6). A long-range correction for the Lennard-Jones interactions was added to all computed solvation free energies. The mean absolute error compared to the experimental data is 1.12 kcal/mol. Our results allow a detailed comparison between the AMBER and CHARMM general force fields and provide a more in-depth understanding of the capabilities and limitations of the CGenFF small molecule parameters.

## Introduction

Alchemical free energy simulations are quickly becoming a routine method in the toolbox of computational chemists.^1−3^ Their predictive capacity depends on (i) the accuracy of the force field used, (ii) the extent of sampling of the relevant regions of phase space, and (iii) the correct setup of the underlying molecular dynamics (MD) simulations.^2,4^ In the case of discrepancies between computed and experimentally measured free energy differences, it is often difficult, if not impossible, to determine the cause of the erroneous results. Especially when studying protein–ligand affinities, incorrect computed binding free energy differences can be caused by either of these three sources of error.

Because biological processes take place in aqueous solution, solvation by water and the accurate calculation of hydration free energies is relevant. The correct representation of a molecule’s interactions with water is a prerequisite for the computational prediction of transfer free energies, i.e., partition coefficients between a polar and non-polar phase, as well as of binding free energy differences. In contrast to proteins, exhaustive sampling is often feasible for small to mid-sized organic molecules. Similarly, the challenge of accurately assigning protomeric states is somewhat simplified compared to the protein environment, given that there are typically few accessible states under physiologically relevant conditions, and their environmental and dynamical dependencies are more readily modeled in isotropic settings. Nevertheless, attention should be paid to the choice of protonation and tautomeric state. Therefore, absolute solvation free energy (ASFE) calculations have served as a sensitive force field accuracy measure.^5,6^

Since the beginning of the century, several studies of increasing scope explored the quality of force fields by comparing the results of ASFE calculations to experiments. Three early examples are the calculation of ASFEs of the amino acid side chain analogues.^7−9^ This work was followed by competitive ASFE prediction challenges involving an increasing number of small organic compounds.^5,10−12^ Already in 2008, Mobley and co-workers computed ASFEs for 504 neutral molecules in implicit solvent. In a subsequent study, they used the AMBER general force field (GAFF)^13^ to calculate ASFEs in explicit solvent for the same set of 504 neutral small organic molecules.^14^ In 2009, Shivakumar et al.^15^ reported the ASFEs of 239 neutral ligands, a test set they also used in later work.^16,17^ In 2011, the Automated force field Topology Builder (ATB) and repository, a web server providing topologies and parameters compatible with the GROMOS force field family,^18^ was used to estimate the ASFE in water for 190 molecules, including the amino acid side chain analogues and various organic molecules from the SAMPL challenges.^19^ The calculation of ASFEs served to validate and refine the ATB as described in subsequent studies.^20,21^ In 2018, Boulanger et al.^22^ introduced General Automated Atomic Model Parameterization (GAAMP) to calculate ASFE for 426 compounds. Similarly to the ATB tool, GAAMP creates charges and parameters based on quantum mechanical calculations, which can be used together with either the GAFF or CHARMM force field.

Calculations as just described require experimental reference data. In 2014, Mobley and Guthrie^23^ established the FreeSolv database. It contains experimentally determined and computed ASFEs for 642 small organic, neutral molecules. The calculations reported in ref (23) were carried out with GROMACS 3.3.1,^24,25^ using the GAFF force field,^13^ explicit water (TIP3P^26^), and AM1-BCC^27,28^ charges. Updates were reported in 2017.^29^ Another source of experimental data is the Minnesota Solvation database,^30^ which also contains solvation free energies for non-aqueous solvents. The late J. Peter Guthrie started the compilation of an even larger collection of experimental solvation free energies of small molecules.^31^ Despite the relatively small size of the molecules in the FreeSolv database compared to typical drugs, the chemical space covered by the database is quite extensive.^23,29^ This makes the database suitable for evaluating the performance of force fields in realistic scenarios involving drug-like molecules.

The FreeSolv database frequently serves as the source of experimental reference values. One recent example is work by Riquelme et al.^32^ who recalculated the entire FreeSolv database with polarized Hirshfeld charges, obtaining a root mean squared error (RMSE) of 2.0 kcal/mol for the whole set. Dodda et al.^33^ calculated the ASFE for a subset of 426 molecules of the FreeSolv testing different charge models together with the OPLS-AA force field.^34^ Computational approaches are not limited to free energy methods based on molecular dynamics (MD). Quantum chemical calculations combined with implicit solvent models are known to predict solvation free energies well.^35,36^ Recently, excellent agreement between computed and experimental ASFE values was obtained using molecular density functional theory.^37,38^ Lately, the FreeSolv database is also used in the field of machine learning to develop and validate models for predicting molecular properties related to solvation and hydration.^39,40^

Large-scale free energy simulations require automated setups. We recently developed and presented a tool, called transformato,^41,42^ for calculating relative solvation and relative binding free energies using the common-core/serial-atom-insertion approach^43^ in a semi-automated manner. Given their importance, we extended the functionality of transformato to the computation of ASFEs. To the best of our knowledge, no systematic study for the compounds in the FreeSolv database has been carried out using the CHARMM general force field (CGenFF).^44−48^ The calculation of the ASFEs for all molecules in the FreeSolv database using CGenFF, therefore, is not only a large-scale test of the new ASFE functionality of transformato but also of wider interest concerning the strengths and weaknesses of this widely used force field.

Specifically, we proceeded as follows. First, we used transformato to calculate the ASFEs for 21 compounds and compared the results to values obtained with the PERT alchemical free energy functionality of CHARMM.^49^ Additional test/validation calculations were carried out to choose the treatment of Lennard-Jones (LJ) interactions and the handling of long-range corrections (LRCs) for the LJ interactions. We then computed the ASFEs of 621 (out of the 642) compounds in the FreeSolv database; see Results and Discussion section for the details on why we were unable to compute ASFEs for some molecules. The results for essentially the complete FreeSolv database are compared to both the experimental values and the results obtained with the GAFF force field. In light of the range of chemical functionalities covered by the FreeSolv database, we briefly analyze the relative strengths and weaknesses of CGenFF and GAFF in their description of a selection of functional groups.

## Methods

### Customizing Transformato for ASFE Simulations

#### Computing Relative Free Energy Differences with Transformato

We developed transformato for the calculation of relative free energy differences. When computing a relative solvation free energy difference between two solutes,^41^ the alchemical transformation from the initial to the final state in vacuum and in aqueous solution passes through an intermediate state, the so-called “common core” (CC). transformato determines a suitable CC by searching for the maximum common substructure of the two molecules. All non-CC atoms are mutated into non-interacting dummy atoms in a stepwise procedure. First, their charges are scaled to zero while maintaining the overall charge of the solute. Next, the LJ interactions of these atoms are removed one by one using the so-called “serial-atom-insertion” (SAI) method.^43^ When turning off the LJ interactions of heavy atoms not present in the CC, each atom is turned off in a separate simulation step. transformato generates all necessary input files (topology, custom parameters for the dummy atoms) so that plain MD simulations can be carried out. No special code, such as soft-core potentials or energy/parameter mixing, is required, making transformato, in principle, independent of the underlying MD program. In practice, OpenMM^50^ is the only fully supported backend presently; CHARMM can be used with some restrictions as well. During each MD simulation, trajectories are saved; these are post-processed to compute the energy differences to all other intermediate states. Finally, the multistate Bennett’s acceptance ratio method (MBAR) as implemented in pymbar(51) is used to obtain free energy differences from these data.^40^ For the full details, we refer the reader to refs (41) and (42).

#### Implementation of ASFE in Transformato

**Figure 1:**
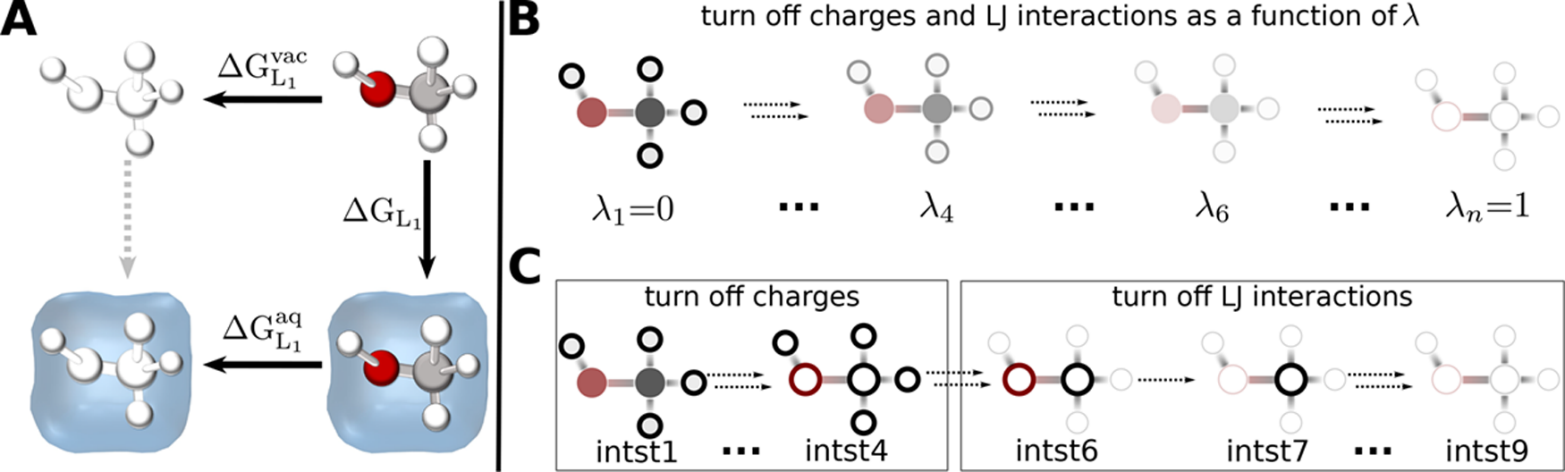
Transformato uses the pathway shown in A for calculating ASFEs. Instead of following the vertical line (Δ*G*_*L*_1__) directly, one follows the two horizontal lines, turning off charges and LJ interactions of the solute, once in solution (aq.) and once in vacuum (vac). In B, a standard approach for calculating ASFEs as, e.g., implemented in the PERT module of CHARMM, is sketched, where non-bonded interactions (charges and LJ interactions) are scaled to zero simultaneously as a function of a continuous coupling parameter λ, typically in *n* = 11 or *n* = 21 steps. In C, the sequence of steps taken by transformato is illustrated, using methanol as the example. During the first four intermediate states (intst1–inst4), the partial charges of the solute are scaled to zero. Afterward, the LJ interactions are scaled to zero, first for all hydrogen atoms in two steps, indicated by the two arrows connecting intst4 with intst6, then for each heavy atom, one after another. The LJ interaction of the last heavy atom is removed in two states.^48^

To calculate the ASFE Δ*G*_*L*_1__, transformato uses the usual thermodynamic cycle shown in Figure 1A. Instead of calculating Δ*G*_*L*_1__ directly, the approach used by transformato follows the horizontal arrows, i.e., all non-bonded interactions of the solute are turned off in the gas phase (Δ*G*_*L*_1__^vac^) and in solution (Δ*G*_*L*_1__^aq^) (an approach referred to as annihilation^52^). The ASFE of interest, Δ*G*_*L*_1__, is obtained according to Δ*G*_*L*_1__ = Δ*G*_*L*_1__^vac^ - Δ*G*_*L*_1__^aq^; see Figure 1A.

In the case of absolute free energy differences, no CC is needed as *all* non-bonded interactions of the solute are turned off. Thus, transformato carries out SAI as described by Boresch and Bruckner^43^ in a fully automated manner. The traditional approach to calculating such free energy differences, e.g., with the PERT module of CHARMM,^49^ is sketched in Figure 1B. The gradual removal of the interactions as a function of a continuous coupling parameter (Figure 1B) should be compared with the transformato/SAI workflow, depicted in panel C of Figure 1: First, the electrostatic interactions of the molecule are turned off. This is achieved by scaling the partial charges of all atoms linearly to zero. By default, four intermediate states are employed, scaling the partial charges of the solute atoms by 1.0, 0.6, 0.3, and 0.0. Next, the LJ interactions of all solute hydrogen atoms are scaled to zero in two steps, scaling *r*_min_ and ε by 0.5 and 0.0. Subsequently, the LJ interactions of the heavy atoms are scaled to zero, one by one, using a single step for each atom. Each heavy atom is thus turned off in a separate intermediate state, except for the last atom, in which case, by default, two intermediate states are used to scale the LJ interactions to zero. As in the case of relative free energy differences, transformato generates all needed files so that for each intermediate state, one can perform a plain MD simulation. Coordinates are saved to disk and are analyzed using the (multistate) Bennett acceptance ratio method (MBAR).^51^

### Workflow—Simulation Details

The FreeSolv database^23,29^ was used as provided on GitHub (https://github.com/MobleyLab/FreeSolv). For each provided SMILES string in the file *database.txt*, a PDB file was created using the Python extension of Open Babel (Pybel).^53^ Missing solute parameters were generated with a stand-alone version of cgenff (v2.5.1), which is based on version 4.6 of the CHARMM general force field (CGenFF).^44−48^ The solutes were placed in cubic simulation boxes with a side length of ≥ 26 Å, which is sufficiently large to be commensurate with the default CHARMM cut-off of 12 Å. Depending on the size of the molecule, the box length may be considerably larger to ensure adequate solvation. The initial side-length of the cubic box, as well as the number of water molecules in the box, are listed for each solute in the Supporting Information. These initial steps were automated with a small utility written in Python, called macha (https://github.com/akaupang/macha), which utilizes CHARMM scripts generated by CHARMM-GUI,^54,55^ as templates. The macha utility wraps these scripts/tools in a package that enables the automatic processing of multiple input molecules into solvated systems that can be simulated with CHARMM or OpenMM. Once basic inputs for simulating a system in the gas phase and in aqueous solution were generated, we invoked transformato to create all intermediate states from full solute–solvent interactions to a solute without any non-bonded interactions as described above. Then, the simulations were run using OpenMM (v7.7).^50^ For each intermediate state, a Langevin dynamics simulation of 5 ns was carried out at 303.15 K; the friction coefficient was set to 1/ps. All simulations were carried out under constant pressure conditions, using a Monte Carlo barostat.^56,57^ Waters were kept rigid throughout the simulation employing the SETTLE^58^ algorithm; the time step was set to 1 fs. Coulomb interactions were calculated using the particle-mesh Ewald (PME) method.^59^ LJ interactions were switched smoothly to zero between 10 and 12 Å with the standard switching function of OpenMM (see eq 1 in the SI). Several simulations were repeated using the force switching function of CHARMM (“vfswitch”),^60^ which can be mimicked using OpenMM; see below for additional details concerning the treatment of LJ interactions. Before each production run, the system was minimized for 500 steps using the L-BFGS minimizer of OpenMM. Simulations of each state were repeated four times with different random initial velocities.

### Post-Processing the Intermediate States

During each of the MD simulations, coordinates were saved to disk every 500 steps, resulting in 10,000 frames per trajectory. The first 25% of each trajectory was discarded as equilibration; the remaining coordinates were used to recompute the energies at all intermediate states. All post-processing was automated by transformato, which then invokes the MBAR functionality of pymbar(51) to compute the free energy differences Δ*G*_*L*_1__^vac^ and Δ*G*_*L*_1__^aq^ (cf. Figure 1A). For each intermediate state *k* and for each configuration sample *x*, the reduced potential *u*(*x*,k) was computed, resulting in a *N* × *K* matrix, where *N* = 7500 is the number of snapshots used and *K* is the number of intermediate states *k* = 1, ..., *K* for a given transformation. The exact number of intermediate states used for each molecule is listed in the Supporting Information. Each set of simulations was repeated four times, using different independent initial velocities (cf. above), so we obtained four statistically independent free energy differences. We used these four values to estimate the statistical error.

### Reference Calculations Using the PERT Module of CHARMM

We utilized the same topology and coordinates (PSF- and CRD files) as for the transformato runs. All calculations were carried out with version c47a1 of CHARMM.^49^ The non-bonded interactions of the solute were turned off in 21 equidistant λ-states. Since we also removed intramolecular non-bonded interactions, a gas phase correction was needed. We set the time step to 1 fs; SHAKE^61^ was applied to the waters only. The PSSP soft-core potential was used to avoid LJ endpoint problems.^49^ In the gas phase, neither LJ nor electrostatic interactions were truncated. In aqueous solution, LJ interactions were switched smoothly to zero between 10 and 12 Å using the potential-based CHARMM switching function.^62^ Electrostatic interactions were computed by PME^59^ (κ = 0.34 Å^–1^; depending on the box size, a 24 × 24 × 24 or 32 × 32 × 32 grid was used for the fast Fourier transforms). In the gas phase, 2 ns simulations were performed (the first 10% was discarded as burn-in), while in solution, we performed 1 ns simulation (again, the first 10% of the simulation was discarded) per λ-state. All calculations were repeated five times. Free energy differences were computed by thermodynamic integration.^63^ The ⟨*dU*/*d*λ⟩_λ_ averages were computed on the fly and extracted from the output files, fitted to a spline function, which was integrated analytically; see Fleck et al.^64^ for additional details.

### Calculation of LJ Long-Range Correction (LRC)

In all simulations in the aqueous phase, the LJ potential was switched off between 10 and 12 Å. Thus, any non-polar, attractive interactions beyond the cut-off radius of 12 Å were omitted. Shirts and co-workers showed that such a truncation can affect binding free energies and outlined how missing dispersion interactions can be accounted for.^65^ In constant volume simulations (NVT), the isotropic LRC for the LJ interactions, which should be sufficient in the case of solute–solvent systems, is a constant term that can be calculated analytically for each molecule after the simulation has been carried out.

For NPT ensembles, the situation is a bit more complex. Shirts et al.^65^ describe several strategies for correcting binding free energy simulations. To account for the fluctuation of the box size during a simulation, at the very least, multiple snapshots of a trajectory have to be analyzed. For each of the 621 compounds, for which we computed the ASFE, we proceeded as follows. In each case, we ran two additional constant pressure MD simulations, one for the fully interacting solute–solvent system, and the other for the water box containing the same number of water molecules as for the native system but with the solute removed. All simulation conditions were identical to what was described above. The simulation length was 3 ns; the first nanosecond was discarded as equilibration. Snapshots were saved every 5 ps (5,000 steps), and their potential energy computed with and without the LRC as outlined in the OpenMM documentation (http://docs.openmm.org/7.7.0/userguide/theory/02_standard_forces.html). Thus, we obtained LRCs Δ*E*_LRC_ for the solute–solvent system and its corresponding water box. By averaging over the 400 snapshots, we obtained averaged values for the solute–solvent system (⟨Δ*E*_LRC_^full^⟩) and for the corresponding water box (⟨Δ*E*_LRC_^water^⟩). The difference ⟨Δ*E*_LRC_^full^⟩ – ⟨Δ*E*_LRC_^water^⟩ is our estimate for the omitted LJ LRC of the solute–solvent interactions. Since these energy differences are relatively noisy, we repeated the above procedure three times, using the average over the three repetitions as the LRC and the standard deviation as its error estimate.

Since an a posteriori correction as just described omits the influence of the LJ long-range interactions on the virial, we ascertained that corrections obtained in this manner are sufficient as follows. We selected nine compounds for which we repeated the full ASFE calculation as set up by transformato with OpenMM’s LRC option turned on. ASFEs obtained in this manner were compared to the values using the approximate a posteriori LRC. In all cases, both approaches gave LRC contributions that agreed within the respective error bars; see Figure S1 in SI.

## Results and Discussion

### Validation of Transformato

The ASFEs of 21 molecules were used to validate the correctness of the results obtained with transformato. Eighteen of these were chosen because we had computed their ASFEs with the PERT module of CHARMM in earlier work.^41,64^ Initially, we assumed that the minor differences in simulation setup and the use of earlier versions of cgenff in our previous work would be irrelevant, but see below. Four out of these 18 molecules are not part of the FreeSolv database, and their experimental ASFEs are not known. In addition, we recomputed the ASFEs with the PERT module of CHARMM for three molecules from the FreeSolv database, for which the values computed with transformato deviated significantly from the experimental results. All 21 compounds are depicted in Figure S2.

The initial comparison of the results, i.e., ASFEs obtained with transformato and the values computed by Fleck et al.^64^ and Wieder et al.^41^ is shown in Figure S3. The RMSE of 0.79 kcal/mol and the mean absolute error (MAE) of 0.46 kcal/mol were surprisingly high. In Figure S3, one sees that most values agree well but that four transformato results deviate by more than 1 kcal/mol from the literature values. One of the deviating ASFEs was obtained for cyclohexa-2,5-dien-1-one from the Fleck et al.^64^ data set (green data points and green box in Figure S3). The three other compounds are 2-methylfuran, 2-cyclopentylindole, and 7-cyclopentylindole, calculated initially by Wieder et al.^41^ (red data points and red box in Figure S3).

While the simulation setup in the earlier studies was very similar to what is described in Workflow—Simulation Details, we realized that force field parameters from different cgenff versions can be quite dissimilar, e.g., for cyclohexa-2,5-dien-1-one, we noted that the partial charges used by Fleck et al.,^64^ derived with cgenff (v2.2), were quite different from those obtained in this study with cgenff (v2.5.1). Thus, we recomputed the ASFE with transformato using the older charges; this reduced the deviation to less than 0.5 kcal/mol. We found similar discrepancies in the parameters, primarily in the partial charges, for the other three problematic molecules. For these cases, we recomputed the ASFEs with PERT as described above, using the cgenff (v2.5.1) force field parameters. We also inspected the partial charges of all other compounds; these were either identical or differed by no more than ±0.02 e.

**Figure 2:**
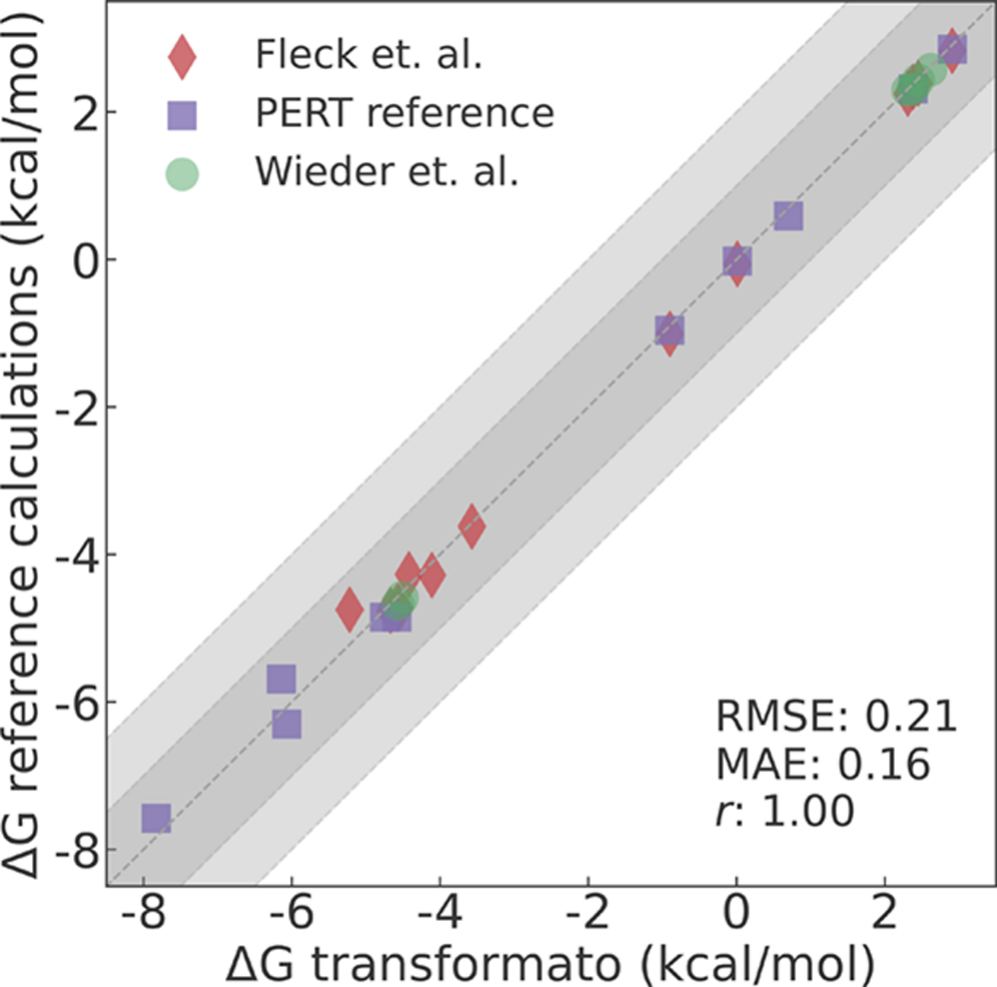
ASFEs computed using transformato either plotted against values extracted from Wieder et al.^41^ (green circles) and Fleck et al.^64^ (red diamonds), or against values recalculated using PERT (purple squares). The overall RMSE and MAE were 0.21 and 0.16 kcal/mol, respectively.

Furthermore, we utilized PERT to compute the ASFEs for three molecules from the FreeSolv database, for which we detected significant discrepancies between the transformato results and the experimental values. The comparison between ASFEs computed with transformato and recomputed free energies using PERT is shown in Figure 2. The RMSE for this refined comparison involving 21 ASFEs was 0.21 kcal/mol, and the MAE was 0.16 kcal/mol. Given that the statistical uncertainty of the computed ASFEs for the 21 molecules is ±0.10 kcal/mol or larger, the agreement between the PERT reference and the transformato results is excellent. The excellent agreement between PERT and transformato results indicates that the initial discrepancies were not caused by transformato but by differences in the versions of the employed force field (mostly in the partial charges).

### Treatment of LJ Interactions

**Figure 3:**
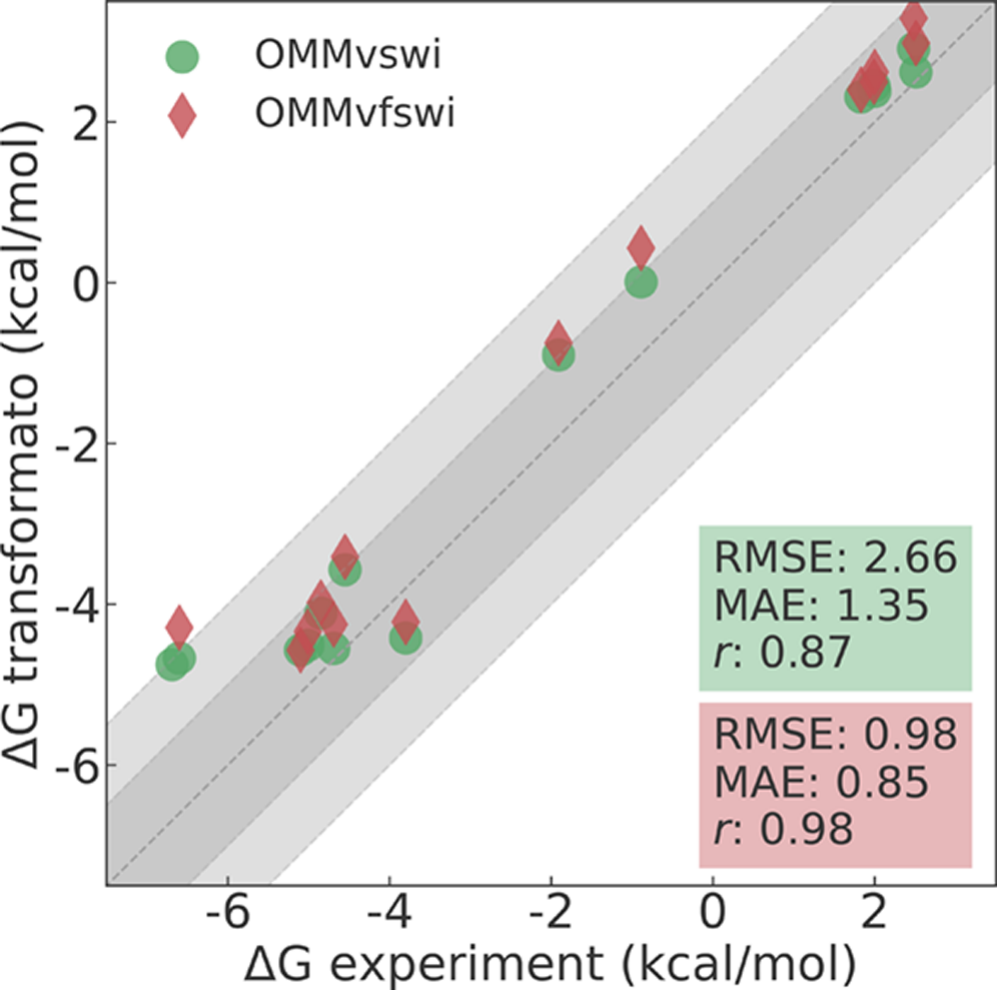
ASFEs calculated once with the OpenMM default switching function (OMMvswi, green dots) and once with the force-switching function (OMMvfswi, red diamonds), plotted against the experimental values. Data points for which no experimental values are available are omitted in this plot but are shown in Figure S4.

While most MD programs treat electrostatic interactions by PME, they often provide several options on how to truncate LJ interactions smoothly at the cut-off distance, e.g., in CHARMM, two switching functions are available, the original potential-based (VSWI, see eq 2 in the SI),^62^ and the newer force-based one (VFSW).^60^ While VFSW should be used with the current family of CHARMM force fields,^66^ the traditional alchemical free energy module PERT of CHARMM only supports VSWI when soft cores are used.^49^ OpenMM natively supports a potential-based switching function, which we will call “OMMvswi.” In addition, CHARMM-GUI provides a custom force routine for VFSW, which we refer to as “OMMvfswi.”^54^ While the functional forms of VSWI and OMMvswi are different, the resulting shapes of the tapering functions are very similar. Therefore, we used OMMvswi in the validation calculations just described. To understand the effects of this particular switching function for LJ interactions on the ASFEs, we used transformato to (re)compute them with OMMvfswi. In Figure 3, we show the results for which experimental solvation free energies are available. One sees that the differences between the OMMvswi (green circles) and OMMvfswi results (red diamonds) are small. In both cases, most ASFEs are too positive compared to experiment. With OMMvfswi, an RMSE of 0.98 kcal/mol and an MAE of 0.85 kcal/mol were obtained. These values reduced slightly to an RMSE of 0.79 kcal/mol and an MAE of 0.66 kcal/mol when using the OMMvswi function (Figure 3). In the SI (Figure S4), we plot the ASFEs obtained with the two treatments of LJ interactions directly against each other. In this plot, we also included solvation free energies of the compounds for which no experimental data are available. The data can be fitted to a regression line *y* = 0.98 *x* + 0.25 (plotted in Figure S4). While the slope is very close to unity, the OMMvfswi results are systematically shifted toward more positive values by +0.25 kcal/mol. A closer examination shows that the difference between the two treatments of LJ interactions increases slightly with the size of the solute, in line with what we observed for the LRC of the LJ interactions (see below).

Thus, at least for the subset of compounds studied, OMMvswi gave results in slightly better agreement with experiment. We, therefore, decided to keep OMMvswi as the truncation method for LJ interactions in the calculations of ASFEs for the full FreeSolv data set. Since we are applying the LRC for the LJ interactions (cf. Calculation of LJ Long-Range Correction (LRC)), the choice of the switching function for LJ interactions used during the simulations should have only negligible effects on the computed ASFEs. In the future, such ambiguities could be avoided using LJPME.^67,68^

### Absolute Solvation Free Energies for the FreeSolv Data Set

#### Difficulties, Challenges, and Failures

With the automated procedure outlined in the Methods section, we obtained ASFEs, which we considered converged and free from major problems, for 621 molecules out of the 642 entries in the FreeSolv database. In 10 cases, the cgenff program failed to parametrize the molecules. These are typically small, simple molecules, such as the carbontetrahalogenides CX_4_, ammonia, or formaldehyde; the list of all 10 molecules can be found in the SI (Table S1). While it would be straightforward to assign force field parameters for these compounds manually, it is presently not possible in an automated manner. Since we want to describe an automated workflow, we did not manually incorporate these molecules even though they might be interesting as test molecules for later force field refinements. The one exception was methane, which also cannot be handled by the cgenff program, since we had force field parameters available in our reference data set (see Validation of Transformato). We also excluded 11 organophosphorodithioates, all of which have a sulfur-phosphorus motif that seems to be handled incorrectly by cgenff/CGenFF. An example of these compounds is shown in Figure S5, together with the Mobley IDs for the other 10 compounds. While we obtained ASFEs for these molecules, the deviation from the experimental values was large in all cases. Upon inspecting the generated force field parameters, we noted that the phosphorus–sulfur double bond was parametrized identically to the P–S single bond (cf. Figure S5), which makes little sense.

In nine out of the remaining 621 cases, we obtained standard deviations between the four individual runs of more than *k*_B_*T* ≈ 0.6 kcal/mol. Upon closer inspection, we noticed that overlap was missing between some adjacent intermediate states. Recalculating these nine ASFEs with 10 ns production time per intermediate state, instead of the default 5 ns, improved the overlap between neighboring states and in all but one cases reduced the standard deviation significantly. For a detailed list of these compounds and the 5 vs 10 ns results, see Table S2.

#### Influence of the LRC

Since we calculated the LRCs as a separate correction, we could analyze how it influenced the overall agreement with the experimental values. The LRC contribution to the ASFE is always negative, ranging from −0.1 kcal/mol for small molecules up to −1.2 kcal/mol for large ones (see Figure S6). We already noted for the validation set that computed solvation free energies tend to be too positive, so the LRC on average improves the agreement with experiment slightly. Indeed, applying the correction improved the overall RMSE by 0.1 kcal/mol and the MAE by 0.2 kcal/mol, compared to the ASFE without LRC. In the remainder of the manuscript, all ASFEs include the LRC. The uncorrected ASFEs for all compounds can be found in the Supporting Information.

#### Comparison between Experiment and Previous Computational Studies

**Figure 4:**
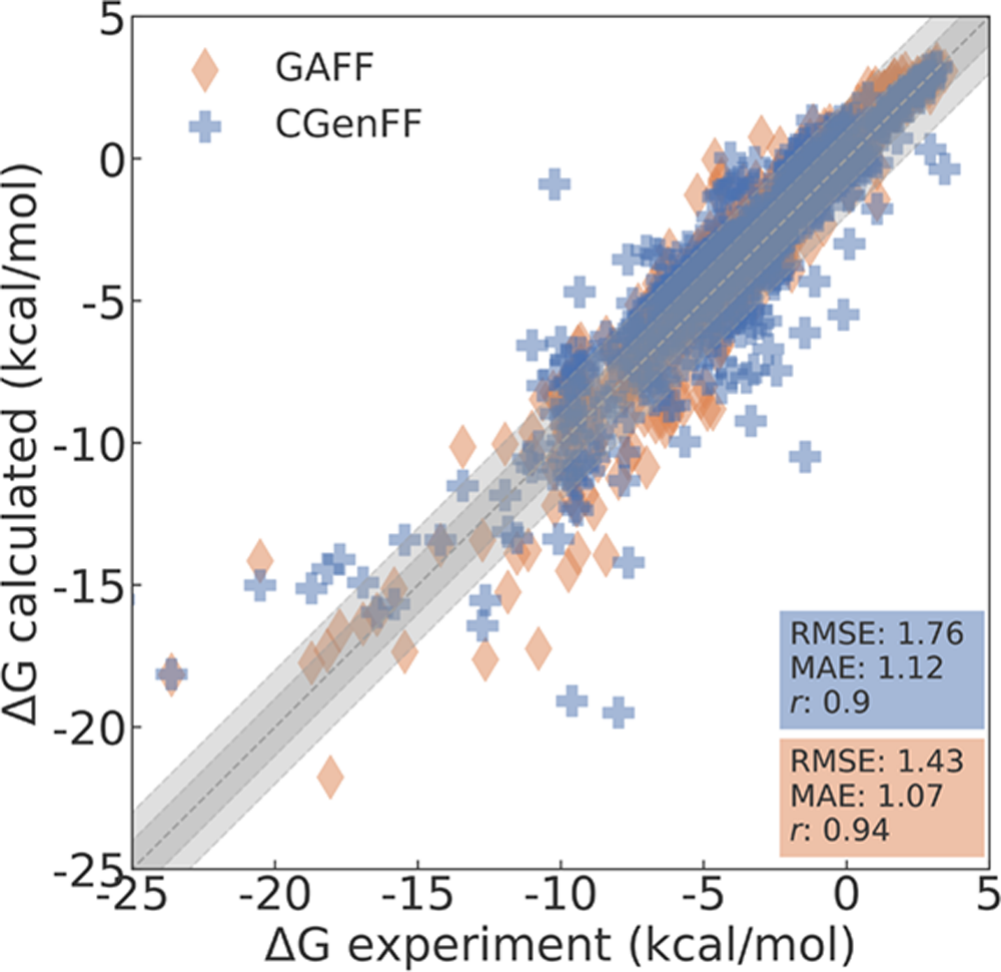
Comparison of the ASFEs for the 621 molecules investigated in this study compared to experimental values from the FreeSolv database.^23^ Results obtained with CGenFF and transformato are marked as blue crosses; results by Mobley and Guthrie^23^ using the GAFF force field are displayed as orange diamonds.

Our results for 621 out of the 642 molecules in the FreeSolv database have an RMSE of 1.76 [1.52,2.02] kcal/mol and an MAE of 1.12 [1.02,1.23] kcal/mol compared to the experimental solvation free energies. The 95% confidence interval is given in brackets, and the bootstrapping procedure is described in the Supporting Information. The Pearson and Spearman correlation coefficients are 0.9 [0.88,0.92] and 0.91 [0.89, 0.93], respectively. The results are plotted in Figure 4 (blue crosses), which also displays the computational results reported in the FreeSolv database (orange diamonds). The detailed results can be found in the Supporting Information.

Our results are in slightly poorer agreement with experiment than the computational results obtained with GAFF.^23^ Using the data as presently reported in the FreeSolv database, the RMSE and MAE for GAFF for the respective 621 molecules are 1.43 [1.31, 1.55] and 1.07 [1.00, 1.15] kcal/mol, respectively. The Pearson and Spearman correlation coefficients both are 0.94 [0.93, 0.95], which is also marginally better. As one can see in Figure 4, our results contain data points that deviate massively from their respective experimental ASFEs. In Table 1, we list the number of molecules deviating by more than a certain threshold from the experimental result. We also include the corresponding numbers for the GAFF results. The numbers in Table 1 confirm that we have poor agreement for more molecules compared to Mobley and Guthrie.^23^

**Table 1:** Number (and Percentage) of Molecules with Large Deviations from the Experimental Results

deviation [kcal/mol]	>2.0	>3.0	>4.0	>6.0
CGenFF	85 (14%)	41 (7%)	20 (3%)	7 (1%)
GAFF^23^	62 (10%)	26 (4%)	13 (2%)	4 (0.5 %)

Aside from the computational results reported in the FreeSolv database, one related large-scale study is the work by Shivakumar et al.^15^ who reported ASFEs for 239 neutral molecules. They compared the commercial version of the CHARMM force field (CHARMm-MSI)^69^ and the standard GAFF force field^13^ with different charge assignments (AM1-BCC/RESP/CHelpG) for the respective compounds. Unfortunately, their molecules are named differently than in this work and no SMILES strings are available, which makes an automated comparison impossible. Performing a spot check, we could identify 101 molecules that are present in both the FreeSolv database and Shivakumar et al.^15^ For this subset, our calculations yielded an RMSE of 1.38 kcal/mol and an MAE of 1.03 kcal/mol, which compares favorably to the results obtained with the commercial CHARMM force field (CHARMm-MSI);^69^ for these 101 molecules, their RMSE was 2.41 kcal/mol, and their MAE was 1.40 kcal/mol. Among the different force fields and charge models they used, a combination of AM1-BCC and the GAFF performed best, with an RMSE of 1.34 kcal/mol and an MAE of 1.05 kcal/mol. This is similar to the performance of the current cgenff/CGenFF combination used in this study.

The validation set for version 3.0 of the ATB^19,20^ contains a significant portion of the FreeSolv database, with 59 molecules excluded due to experimental uncertainties of 1 kcal/mol or more.^21^ This resulted in an MAE of 1.00 kcal/mol and an RMSE of 1.5 kcal/mol. When excluding compounds with experimental uncertainties greater than 1.0 kcal/mol, the performance of the CGenFF results presented here is comparable, with an MAE of 1.08 kcal/mol and an RMSE of 1.68 kcal/mol.

The largest too negative deviation was observed for cyanuric acid, where we missed the experimental ASFE (−18.06 kcal/mol) by 11.59 kcal/mol, with a calculated ASFE of −29.65 kcal/mol. In the opposite direction, we obtained the most positive wrong result for β-glucose, −15.51 kcal/mol instead of the experimental value of −25.47 kcal/mole. Both molecules were part of the SAMPL2 challenge^5^ and were among those compounds having the largest variation in results during the competition. Cyanuric acid may adopt multiple tautomeric forms. Initially, we simulated the *all-oxo* form based on the SMILES code provided in the FreeSolv database as it is believed to be the dominant form in solution.^70,71^ However, Pérez-Manríquez et al.^72^ suggest that the aromatic enol form may partially exist in aqueous solution as well. Thus, we also calculated the ASFE for this tautomeric form, obtaining a value of −7.27 kcal/mol. For glucose, the organizers of the SAMPL2 challenge pointed out the high flexibility of the sugar and argued that its polarity, and hence, its ASFE, may change considerably upon a conformational change. Thus, in both cases, the origin of the large error may not be caused by the force field alone.

#### Performance for Different Functional Groups

**Figure 5:**
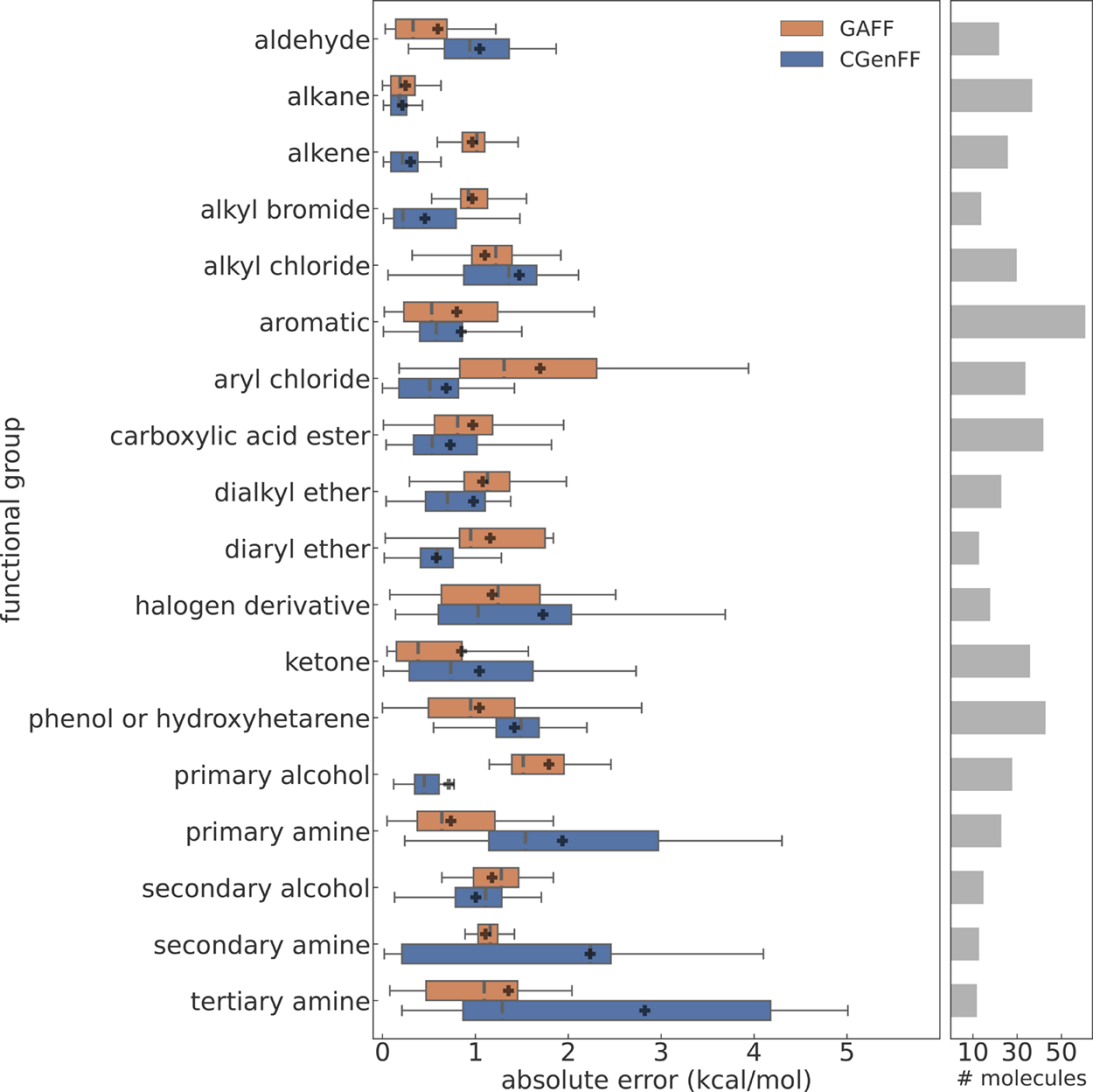
Box-like plot of the ASFE results for groups of molecules sharing the same chemical functionality; results are only shown when there are at least 10 molecules belonging to a group in the database. The classification into functional groups follows the original work by Mobley and Guthrie.^23^ Left: the absolute error compared to the experiment, obtained with transformato/CGenFF (blue, this work) and GAFF^23^ (orange) are shown in a box plot-like manner. For each calculated ASFE, the absolute deviation from its experimental value is calculated, grouped and assigned to the corresponding functional group. The black crosses indicate the mean absolute error (MAE) for a particular group. The whiskers indicate the molecules with minimal and maximal deviation from the experimental ASFE. The bars depict the range from the first to the third quantile of the MAE values. The median absolute error is indicated as a thin, dashed vertical line. Right: the gray bars indicate the number of molecules belonging to a group.

We categorized the molecules based on their chemical functionalities using the *groups.txt* file from the FreeSolv repository available on GitHub (https://github.com/MobleyLab/FreeSolv). While many compounds in the data set exhibit a high degree of polyfunctionality, there are also large groups of monofunctional molecules. To avoid double-counting polyfunctional compounds, we utilized only the first category provided in the *groups.txt* file to assign the molecules to their respective categories. The assignment used is listed in the Supporting Information. Proceeding in this manner results in some ambiguity, e.g., our category “amine” contains both aliphatic and aromatic compounds. We consider this acceptable for a quick survey, and the same criteria were applied to our results, as well as to those of Mobley and Guthrie.^23^ Furthermore, since the number of molecules in the FreeSolv database is not too large to begin with, more detailed classification attempts quickly lead to categories consisting of only very few molecules. Figure 5 summarizes our analysis. For all functional groups, for which there are at least 10 molecules in the database, we plot the absolute error obtained with transformato/CGenFF (blue) against the GAFF results (orange).^23^ Based on the MAE (black crosses in Figure 5), GAFF outperformed CGenFF for nine out of the 18 investigated functional groups; i.e., for these groups, the use of GAFF led to a lower MAE. Conversely, CGenFF yielded a lower MAE for the remaining nine functional groups. In Figure 5, one sees that CGenFF performs notably worse than GAFF for the primary, secondary, and tertiary amines, the latter having an MAE (black cross) of 2.6 kcal/mol; the corresponding RMSE was 3.2 kcal/mol. Overall, tertiary amines were the chemical functionality for which calculations with CGenFF presented the largest deviations from the experimental results. Note, though, that in terms of the median absolute error (gray dashed line), the performance of CGenFF is much closer to that of GAFF. Primary and secondary amines were the other two functional groups for which the MAE obtained with CGenFF was > 2 kcal/mol. For the halogen derivatives and alkyl chlorides, CGenFF also gave results in poor agreement with experiment (MAE > 1.5 kcal/mol). Examples of functional groups for which GAFF performed worse are aryl chlorides and primary alcohols. In these two cases, GAFF resulted in MAEs > 1.5 kcal/mol, significantly higher than the MAEs obtained with CGenFF. CGenFF also performs significantly better for diaryl ethers, whereas, for the dialkyl ethers, both force fields give similar results.

We conducted similar analyses with the other used metrics (RMSE and the Pearson correlation coefficients); see Figure S7. The results for the Pearson correlation coefficient contained one unexpected data point. For the diaryl ethers, the GAFF results show a negative Pearson’s *r*, possibly indicating one or more erroneous entries in the database. It should be noted that for this group, CGenFF also has an *r* value of only 0.6. In line with the MAE results, the CGenFF *r* values for primary, secondary, and tertiary amines are low as well; for all other chemical groups *r*(CGenFF) ≥ 0.8.

## Conclusions

### Advantages of Transformato

**Figure 6:**
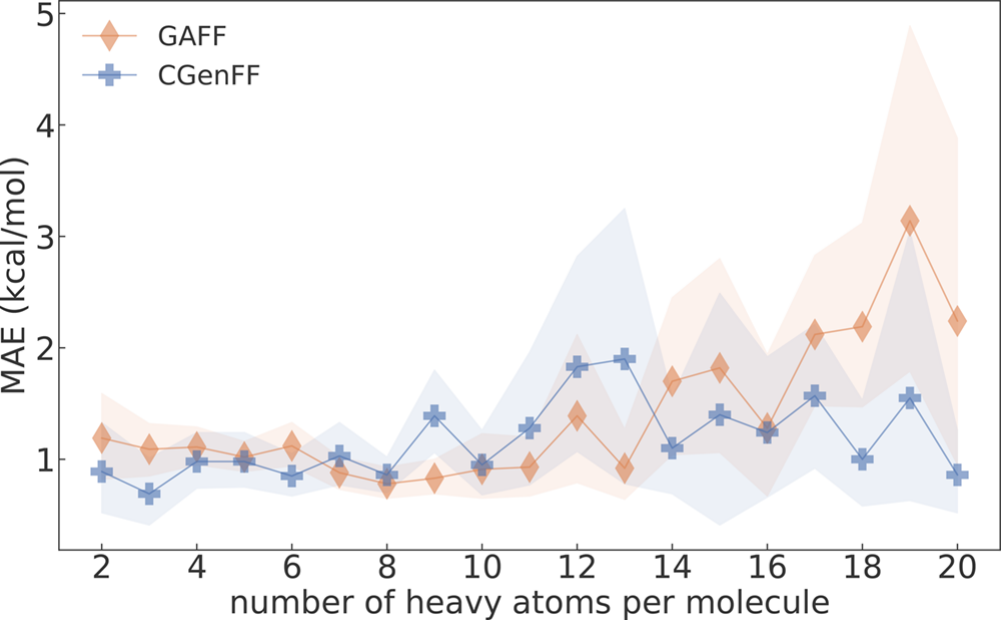
MAEs for the calculated ASFEs grouped by the number of heavy atoms. Values denoted GAFF were obtained using a standard lambda protocol with 21 lambda states. Values denoted CGenFF were obtained using transformato, with different numbers of intermediate states, depending on the size of each molecule. The shaded region surrounding the lines corresponds to the upper and lower bounds of the bootstrapped error for molecules that have the same number of heavy atoms.

The present results, as well as those of refs (41) and (42), demonstrate the utility of transformato in setting up and carrying out large-scale free energy calculations. By relying on SAI, the underlying MD program does not need support for special purpose code, such as soft-core potentials. Not counting the initial validation and preliminary tests for some subsets, we were able to compute the 621 ASFEs in 4 weeks, utilizing on average 15 consumer-grade GPUs (the fastest ones being NVIDIA RTX2080 cards). Furthermore, since transformato provides self-contained inputs for each intermediate state, computations can be easily distributed across as many nodes as there are available. For a medium-sized molecule from the FreeSolv database with seven heavy atoms (e.g., toluene), 15 intermediate states are necessary. When running them in parallel with the local resources just described, a simulation of one intermediate state takes approximately 20 minutes. The post processing of the trajectories takes another 20 minutes; thus, on this small cluster, the calculation of an ASFE requires less than an hour of wall time. A peculiarity of SAI is that the number of intermediate states depends on the size of the alchemical region of the solute, in particular, the number of non-hydrogen atoms. This contrasts with most standard free energy simulation protocols, in which a fixed number of intermediate λ-states, e.g., 11 or 21, is used for all alchemical transformations. At first glance, this is a downside since the larger a molecule, the longer it takes to compute its solvation free energy simply because more intermediate states are necessary. However, this intrinsic adaptiveness also has advantages. First, for smaller molecules, e.g., ethane or methanol, typical protocols with 21 λ-states are inefficient. Conversely, these 21 λ-states may be not enough for large solutes. In Figure 6, we plot the MAEs sorted by the number of solute heavy atoms. The present results (blue) are compared to the ASFEs from Mobley and Guthrie,^23^ who used a 21 λ-states protocol. For molecules with 14 heavy atoms or more, the MAEs of the transformato results are lower than those of Mobley and Guthrie.^23^ Thus, transformato automatically imposes less costly protocols for small(er) molecules and more expensive ones for large(r) compounds; the data in Figure 6 indicate that this extra effort is well spent.

### cgenff/CGenFF for ASFE

Overall, the use of cgenff/CGenFF leads to a slightly worse agreement with experimental ASFEs compared to GAFF. As one can see from Table 1, this is caused by a relatively small number of compounds for which the computed ASFEs are off by 2 or more kcal/mol. There is no trivial way to relate a wrong free energy difference to particular force field parameters (though there are attempts such as “Time Machine” (https://github.com/proteneer/timemachine)), and a systematic failure analysis is out of scope for this study. At the same time, it was straightforward to identify a chemical functionality, in particular amines, for which CGenFF tends to perform poorly (cf. Figure 5). Although occasionally used,^73^ the comparison of computed ASFEs to experimental data is not a routine part of parameter optimization for the additive CHARMM force field family. In light of this, we consider the agreement between computed and experimental ASFEs quite satisfactory.

Users of CGenFF should, however, keep some additional cautions in mind. Force field parameters generated by the cgenff program are different depending on the version used. Similarly, for a compound that is part of CGenFF’s template set (i.e., which is explicitly present in the topology file top_all36_cgenff.rtf), one may obtain different partial charges when processing the molecule with cgenff compared to the charges found in the template topology file. Since the parameters generated by cgenff are based on a machine-learning model, the resulting assigned charges and bonded parameters can vary as the training set for the force field is extended.^44,74^ Keeping this in mind, the above observations are the expected behavior. Nevertheless, some differences in parameters we encountered during the validation phase were unexpectedly drastic, which caused some confusion, e.g., for 2-methylfurane, there are differences in partial charges of > 0.2 e between the two cgenff/CGenFF versions, changing the computed ASFE by almost 2 kcal/mol. The need for strict version control of parameters and keeping the version of cgenff constant during a project might need to be better communicated.

## Summary

In conclusion, the implementation of transformato establishes a scalable solution for calculating ASFEs. Separating the “alchemical setup and post-processing” from running the underlying MD simulations is beneficial as the two functionalities can be optimized independently. The computational efficiency of transformato is also a step toward democratizing access to extensive testing of force fields, empowering even smaller research entities with limited resources to perform ASFE calculations for the FreeSolv database. The results obtained offer insights in the limitations of the cgenff/CGenFF small molecule parameter set for calculating ASFEs and may serve as a starting point for further development of the CGenFF force field.

## Data Availability

All plots shown in this paper were produced using the Jupyter-notebook available on GitHub (https://github.com/JohannesKarwou/notebooks/blob/main/freeSolvSummary.ipynb). The notebook also contains the calculations of all statistics reported in this paper (RMSE, MAE, Pearson correlation, and Spearman’s rank correlation), as well as the corresponding bootstrapped errors. Transformato version used in this work (release v0.3): https://github.com/wiederm/transformato. Macha version used in this work (release v0.0.1): https://github.com/akaupang/macha.

## Supporting Information Available

Supporting figures and tables. Figure S1: comparison of two approaches to compute the LJ LRC; Figure S2: molecules for which ASFEs with comparable simulation setups and force field parameters have been reported in the literature; Figure S3: initial comparison between results from transformato (TF) and reference results from the literature before ensuring that identical force field parameters were used; Figure S4: comparison of ASFEs calculated with TF when using two different switching functions for the LJ interactions; Figure S5: one of the 11 organophosphorodithioates for which we report no ASFEs (Mobley ID: 7754849); Figure S6: average long-range correction as a function of the number of atoms per molecule, including hydrogens, are shown as blue squares; Figure S7: expanded statistical measures for the results reported in this study (blue) and by Mobley and Guthrie; Table S1: all molecules for which the cgenff program failed to provide parameters; and Table S2: molecules, for which the standard deviation (std) of the ASFE (dG TF) was >0.6 kcal/mol when using the default simulation length per state of 5 ns (PDF)
Supporting spreadsheet. Entries labeled *dG (TF)* are the direct results calculated with transformato, entries labeled *dG (TF) lrc* are the long-range corrected ASFE, i.e., the final results on which our analysis and discussion are based; entry *std* lists the standard deviation between the four individual runs; file further provides the number of intermediate states used for each ASFE calculation (*nr intst*), and the initial box size and number of water molecules for each solute (*box length* and *nr waters*); and functional group used for the analysis of the respective molecule can be found in the *functional group* column (XLSX)
Force field parameters for each solute generated by cgenff (ZIP)
