# Supplementary material for: Calculations of Absolute Solvation Free Energies with Transformato—Application to the FreeSolv Database Using the CGenFF Force Field
Source: J Chem Theory Comput. 2023 Aug 24;19(17):5988–98. doi: 10.1021/acs.jctc.3c00691 (PMC10500982; doi:10.1021/acs.jctc.3c00691)
Supplement: Supplementary file 1 — ct3c00691_si_001.pdf [file ct3c00691_si_001.pdf]

Calculations of absolute solvation free energies with `transformato`— application to the FreeSolv database using the CGenFF force field

Johannes Karwounopoulos,[†,‡] Åsmund Kaupang,[¶] Marcus Wieder,[§] and Stefan Boresch[*,†]

†*Faculty of Chemistry, Institute of Computational Biological Chemistry, University of Vienna, Vienna, Austria*

‡*Vienna Doctoral School of Chemistry (DoSChem), University of Vienna, Vienna, Austria*

¶*Department of Pharmacy, Section for Pharmaceutical Chemistry, University of Oslo, 0316 Oslo, Norway*

§*Department of Pharmaceutical Sciences, Pharmaceutical Chemistry Division, University of Vienna, Althanstrasse 14, 1090 Vienna, Austria*

E-mail: stefan@mdy.univie.ac.at

Bootstrapping statistics

For all statistical measures (root mean squared error (RMSE), mean absolute error (MAE), Pearson correlation (r), and Spearman's rank correlation reported in this work, we report error estimates obtained by bootstrapping. Values were selected randomly with replacement from the original dataset 1,000 times, and all statistical measures were computed for each random selection. The resulting resampled metrics were stored in a list. Subsequently, the 95% confidence interval was computed by determining the 2.5th and 97.5th percentiles of the resampled metrics. These were used as upper and lower bounds for the statistical measures.

Potential-based switching functions in OpenMM and CHARMM

Default switching function in OpenMM

The standard switching function of OpenMM referred to as OMMvswi in this manuscript is defined as follows *:

$$S(x) = 1 - 6x^5 + 15x^4 - 10x^3 \tag{1}$$

where $x = \frac{(r - r_{\text{switch}})}{(r_{\text{cutoff}} - r_{\text{switch}})}$. It decreases from 1 at $r = r_{\text{switch}}$ to 0 at $r = r_{\text{cutoff}}$.

Switching function in CHARMM

Since it is somewhat hidden in a relatively old publication, we also give the equations for the vswitch function of CHARMM as described originally by **?**:

$$S(x, x_{on}, x_{off}) = \frac{(x_{\text{off}} - x)^2 (x_{\text{off}} + 2x - 3x_{\text{on}})}{(x_{\text{off}} - x_{\text{on}})^3} \tag{2}$$

*http://docs.openmm.org/7.7.0/userguide/theory/02_standard_forces.html

Note that this function is used as $S(\mathbf{r^2}, \mathbf{r^2_{on}}, \mathbf{r^2_{off}})$, where r is the distance between the two particles, r_{off} the cutoff distance, and r_{on} the distance where the switching region starts. The function decreases from 1 at $r = r_{\text{on}}$ to 0 at $r = r_{\text{off}}$.

Figures

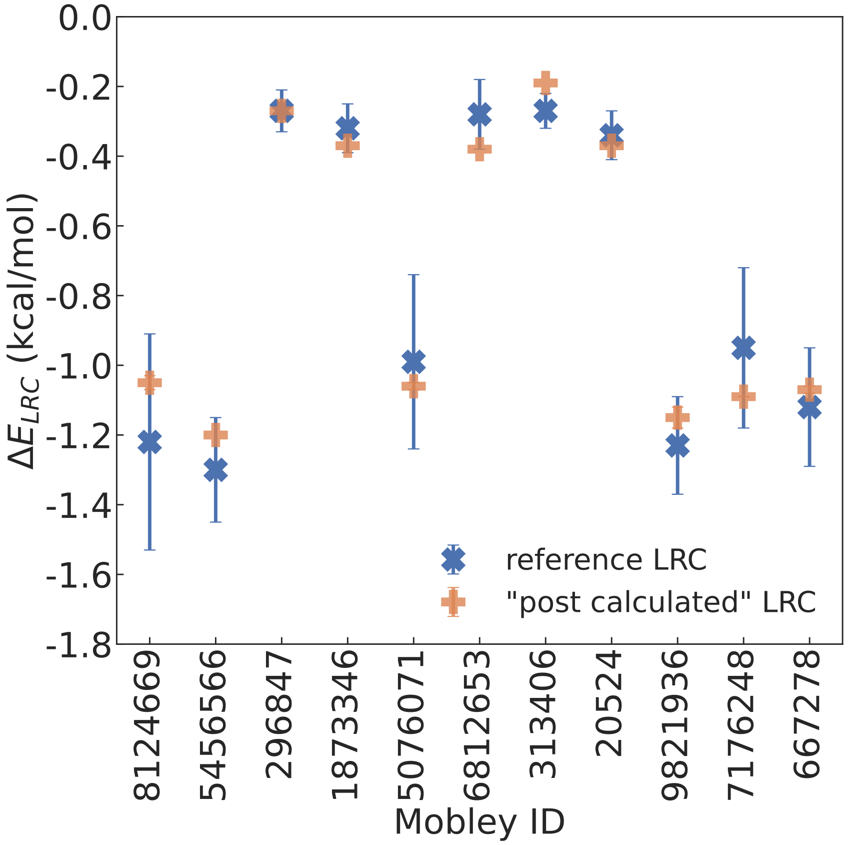

Figure S1: Comparison of two approaches to compute the Lennard-Jones long-range correction (LRC), For all compounds studied in this work, the LRC was estimated in a post-processing step ("post-calculated LRC", orange crosses); cf. main manuscript. For eleven compounds, the LRC was also calculated more correctly as follows: For each of the molecules, the ASFE was recomputed from scratch with OpenMM's LRC set to active during all MD simulations, thus including the LRC to the virial. The difference between the ASFE including the full isotropic LRC computed in this manner and the ASFE without LRC is referred to as "reference LRC" and plotted with a blue x. As one can see, the two LRCs agree reasonably well in all cases.

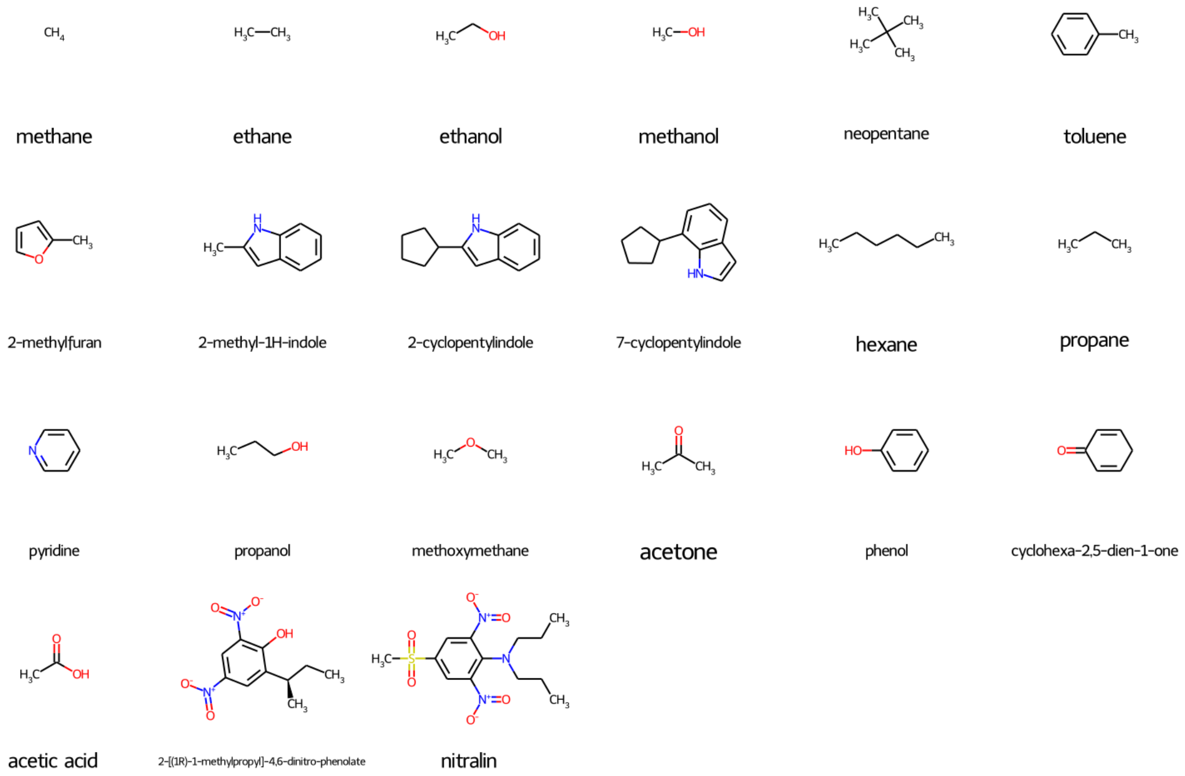

Figure S2: Molecules for which ASFEs with comparable simulation setups and force field parameters have been reported in the literature.[1,2] This set was augmented by three additional molecules (last row) taken from the FreeSolv database: acetic acid (Mobley ID: 2725215), 2-[(1R)-1-methylpropyl]-4,6-dinitro-phenolate (Mobley ID: 3274817) and nitralin (Mobley ID: 725215).

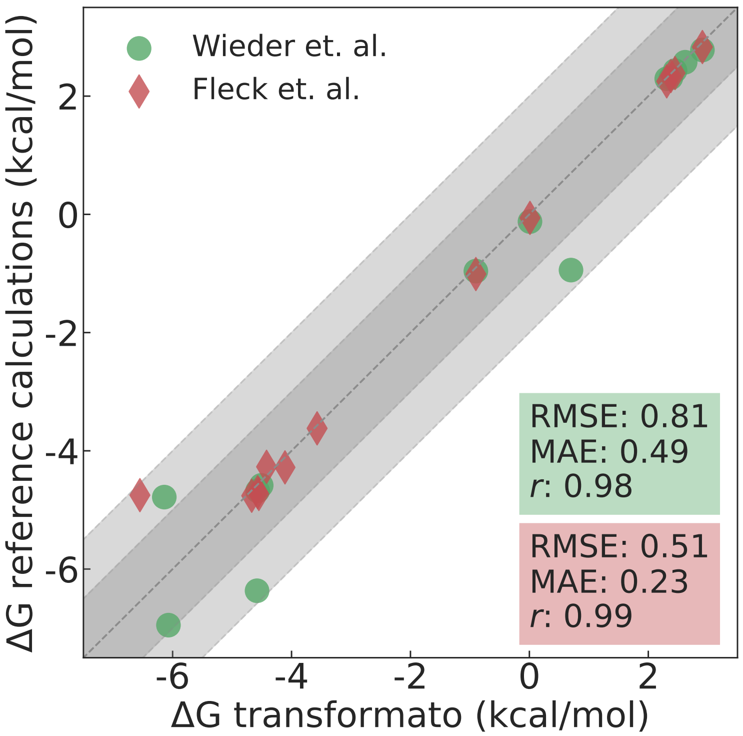

Figure S3: The initial comparison between results from `transformato` (TF) and reference results from the literature[1,2] *before* ensuring that identical force field parameters were used.

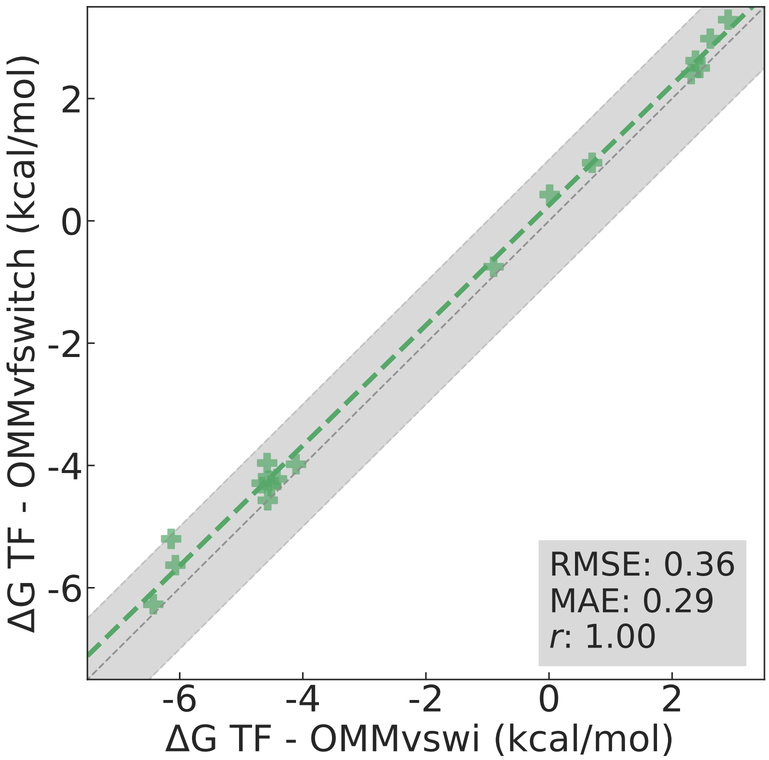

Figure S4: Comparison of ASFEs calculated with `transformato` (TF) when using two different switching functions for the Lennard-Jones interactions (OMMvswi and OMMvfswi); cf. the main manuscript. Values for all compounds in Fig. S2 – including the ones for which no experimental values are available – are shown. The green dashed line represents the linear regression line $y = 0.98x + 0.25$.

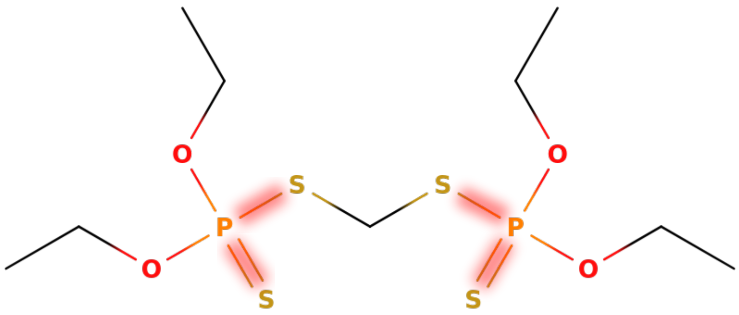

Figure S5: One of the 11 organophosphorodithioates for which we report no ASFEs (Mobley ID: 7754849). The phosphorus-sulfur bonds for which `cgenff` provided dubious force field parameters are highlighted in red. The Mobley IDs of the other compounds with the same motif are: 1770205, 1922649, 1849020, 2518989, 1849020, 2518989, 5393242, 8916409, 9281946, 7326706.

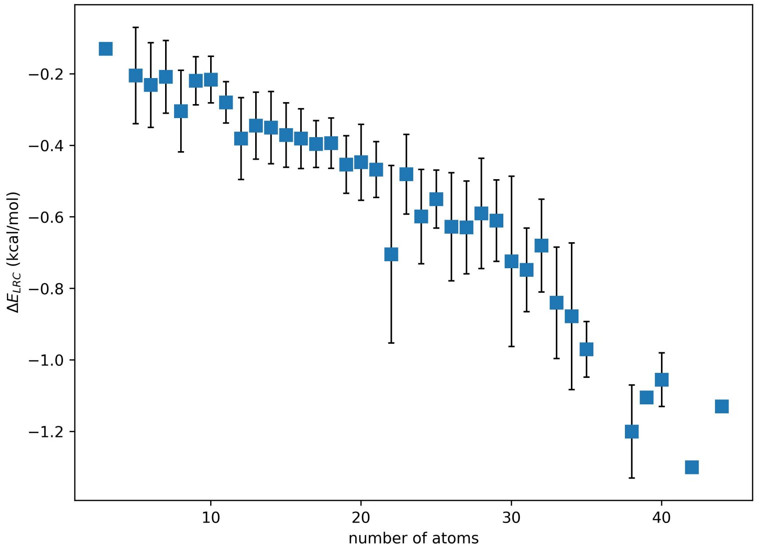

Figure S6: The average long-range correction ΔE_{LRC} as a function of the number of atoms per molecule, including hydrogens, are shown as blue squares. The spread of the correction for all molecules consisting of the same number of atoms are shown as error bars. If there are less than 3 molecules with the same number of atoms, no error bars are shown.

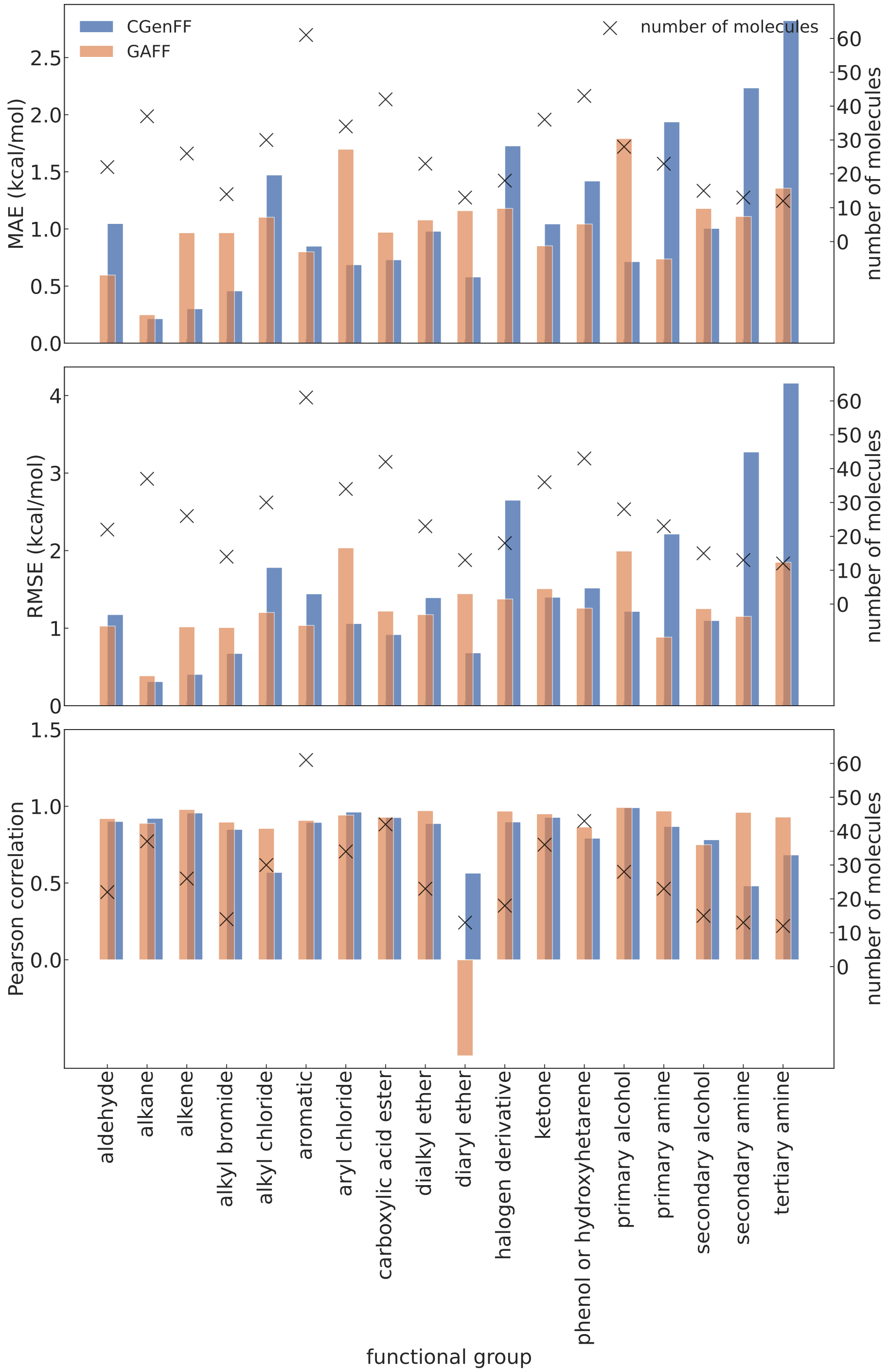

Figure S7: Expanded statistical measures for the results reported in this study (blue) and by Mobley and Guthrie[3] (orange). From top to bottom: mean absolute errors (MAEs), root-mean-squared errors (RMSEs) and Pearson correlations. The molecules are classified according to their primary functional group. In each plot, the crosses, corresponding to the second y-axis on the right, indicate the number of molecules belonging to each group.

Tables

Table S1: All molecules for which the `cgenff` program failed to provide parameters.

Mobley ID	smiles	IUPAC name
2146331	C=O	formaldehyde
6359135	C(Cl)(Cl)(Cl)Cl	carbon tetrachloride
7578802	C(Br)(Br)Br	bromoform
676247	C(F)(F)(F)F	tetrafluoromethane
8311321	C(F)(F)Cl	chloro-difluoro-methane
5631798	N	ammonia
7732703	C(F)(F)(F)Br	bromo-trifluoro-methane
2996632	C(Cl)(Cl)Cl	chloroform
9571888	C1[C@@H]2[C@H](COS(=O)O1)[C@@]3(C(=C([C@]2(C3(Cl)Cl)Cl)Cl)Cl)Cl	endosulfan alpha

Table S2: Molecules, for which the standard deviation (std) of the ASFE (dG TF) was > 0.6 kcal/mol when using the default simulation length per state of 5 ns. Increasing the simulation length per state to 10 ns (dG TF (10 ns)) reduced the standard deviation (std) to < 1 kcal/mol in all cases.

Mobley ID	exp	dG TF (5 ns)	std	dG TF (10 ns)	std
2613240	-5.33	-1.85	1.64	-4.63	0.66
5200358	-9.84	-10.32	1.51	-9.70	0.82
3265457	-7.78	-8.66	1.28	-9.61	0.45
6935906	-2.49	-3.07	1.16	-3.14	0.58
4587267	-23.62	-17.05	1.01	-17.43	0.51
6688723	-6.44	-7.08	0.98	-6.99	0.50
3515580	-5.94	-3.66	0.84	-3.3	0.52
8705848	-3.22	0.14	0.75	0.23	0.3
8916409	-8.15	-16.58	0.6	-15.8	0.7

References

(1) Wieder, M.; Fleck, M.; Braunsfeld, B.; Boresch, S. Alchemical free energy simulations without speed limits. A generic framework to calculate free energy differences independent of the underlying molecular dynamics program. *J. Comput. Chem.* **2022**, *43*, 1151–1160.

(2) Fleck, M.; Wieder, M.; Boresch, S. Dummy Atoms in Alchemical Free Energy Calculations. *J. Chem. Theory Comput.* **2021**, *17*, 4403–4419.

(3) Mobley, D. L.; Guthrie, J. P. FreeSolv: a database of experimental and calculated hydration free energies, with input files. *J. Comput. Aided Mol. Des.* **2014**, *28*, 711–720.
